# Tuneable Red and Blue Emission of Bi^3+^-Co-Doped SrF_2_:Eu^3+^ Nanophosphors for LEDs in Agricultural Applications

**DOI:** 10.3390/nano14201617

**Published:** 2024-10-10

**Authors:** Jovana Periša, Sanja Kuzman, Aleksandar Ćirić, Zoran Ristić, Željka Antić, Miroslav D. Dramićanin, Bojana Milićević

**Affiliations:** Centre of Excellence for Photoconversion, Vinča Institute of Nuclear Sciences—National Institute of the Republic of Serbia, University of Belgrade, 11000 Belgrade, Serbia; jburojevic@vinca.rs (J.P.); sanja.kuzman@vinca.rs (S.K.); aleksandar.ciric@ff.bg.ac.rs (A.Ć.); risticz@vinca.rs (Z.R.); zeljka.antic@vinca.rs (Ž.A.)

**Keywords:** lanthanide luminescence, phosphors, LEDs for agricultural application, Eu^3+^, Bi^3+^

## Abstract

Tunable blue/red dual-emitting Eu^3+^-doped, Bi^3+^-sensitized SrF_2_ phosphors were synthesized utilizing a solvothermal-microwave method. All phosphors have cubic structure (*Fm*-*3m* (225) space group) and well-distinct sphere-like particles with a size of ~20 nm, as examined by X-ray diffraction and transmission electron microscopy. The diffuse reflectance spectra reveal a redshift of the absorption band in the UV region as the Bi^3+^ concentration in SrF_2_: Eu^3+^ phosphor increases. Under the 265 nm excitation, photoluminescence spectra show emission at around 400 nm from the host matrix and characteristic orange ^5^D_0_ → ^7^F_1,2_ and deep red ^5^D_0_ → ^7^F_4_ Eu^3+^ emissions. The red emission intensity increases with an increase in Bi^3+^ concentration up to 20 mol%, after which it decreases. The integrated intensity of Eu^3+^ red emission in the representative 20 mol% Bi^3+^ co-doped SrF_2_:10 mol% Eu^3+^ shows twice as bright emission compared to the Bi^3+^-free sample. To demonstrate the potential application in LEDs for artificial light-based plant factories, the powder with the highest emission intensity, SrF_2_: 10Eu, 20 Bi, was mixed with a ceramic binder and placed on top of a 275 nm UVC LED chip, showing pinkish violet light corresponding to blue (409 nm) and red (592, 614, and 700 nm) phosphors’ emission.

## 1. Introduction

Plants require light for photosynthesis, morphogenesis, physiological reactions, and the buildup of bioactive compounds [1]. The usage of LEDs (light-emitting diodes) in plant cultivation has markedly increased in recent years due to their cool emitting surface, extended longevity, and customizable spectrum composition aligned with the absorption wavelengths of plant photoreceptors [2,3]. Plants absorb light for photosynthesis in the 300–800 nm wavelength range, although different plant photoreceptors have varying absorption properties [4].

The photosynthetically active radiation (PAR) spectrum, which spans from 400 to 700 nm, is crucial for plant growth as it encompasses the wavelengths most effective for photosynthesis. Plants primarily absorb blue and red light to drive this process, and recent research has demonstrated the importance of deep-red light for the growth of leafy plants [5]. Blue light supports vegetative growth and leaf development, promoting strong, compact plants with efficient photosynthesis. Red light is essential for flowering and fruiting, influencing plant morphology and the timing of these developmental stages. Although red light is crucial for leaf expansion, blooming, and overall plant growth, some studies suggest that plants under red light can achieve higher dry weight compared to those exposed to mixed red and blue light [6,7]. Additionally, blue light may negatively impact chlorophyll concentration [2]. Adding a specific amount of blue to red light in LEDs for agricultural applications is crucial for balancing the effects on plant photoreceptors. This combination helps avoid the drawbacks of using only red or blue light, ensuring a more effective interaction with both blue-absorbing cryptochrome and red-absorbing phytochrome. This balance is essential for promoting optimal plant health and growth [8]. Researchers have found that using a significant amount of blue light joint with the red one inhibits growth and leaf expansion, making it an effective strategy for controlling height in various bedding plants while preserving flowering and post-harvest quality [9,10]. *Herbaceous perennial* plants grown under a 50:50 blue/red LED light combination demonstrate improved root biomass and stem elongation, which minimizes damage during transportation and transplantation [11]. Incorporating blue light into a dominant red-light environment may inhibit the rapid dehydration of cuttings; blue light promotes stomatal opening and trichome production, maintaining photosynthesis while preventing transpiration [12]. Therefore, the development of suitable phosphors that can absorb blue or UV light and emit tunable blue-red light has evolved into an imperative in crop production.

Eu^3+^ with the 4*f*^6^ electronic configuration is a lanthanide red-emitting ion that exhibits orange, red, and deep red emission corresponding to transitions from the excited ^5^D_0_ level to the ground ^7^F*_J_* (*J*  =  1, 2, 3, and 4) levels [13]. On the contrary, Bi^3+^ ions can act as a sensitizer, increasing phosphor absorption. The effective energy transfer processes from Bi^3+^ to Eu^3+^ have been explored in the following materials, which may be used in general illumination or plant growth LEDs: BaSc_2_O_4_: Li^+^, Eu^3+^, Bi^3+^ [14], Na_4_CaSi_3_O_9_: Bi^3+^, Eu^3+^ [15], BaGd_2_O_4_: Bi^3+^, Eu^3+^ [16], Ba_9_Lu_2_Si_6_O_24_:Bi^3+^, Eu^3+^ [17], and KBaYSi_2_O_7_: Bi^3+^, Eu^3+^ [18], Lu_2_GeO_5_: Bi^3+^, Eu^3+^ [19], Gd_3_GaO_6_: Bi^3+^, Eu^3+^ [20], etc. By selecting the appropriate host, the emission spectra of Bi^3+^–Eu^3+^ co-activated luminescent materials can be fine-tuned to achieve blue-red double emission, effectively aligning with the absorption spectra of plant photoreceptors such as cryptochrome and phytochrome. Among various classes of inorganic host materials, fluoride-based phosphors exhibit numerous outstanding properties, including a broad optical transmission range, anionic conductivity, low phonon energy, high resistivity, and reduced nonradiative relaxation of excited states, which ultimately enhances radiative emission [21,22]. Moreover, they can be prepared by simple, environmentally friendly, and HF-free methods [21,22,23,24,25,26,27]. Mancebo et al. found that Bi^3+^ sensitization enhanced Eu^3+^ emission in LaF_3_ by more than one order of magnitude because the bismuth ion in this host lattice improves the material’s X-ray attenuation capacity [25]. Luo et al. also showed that Bi^3+^-sensitized NaGdF_4_: Eu^3+^ nanocrystals for white LEDs exhibit increased red emission through co-doping with Bi^3+^ [26]. In addition to fluoride materials with enhanced red emission, tunable blue-red double-emitting phosphors that correspond to the absorption of cryptochrome and phytochrome plant photoreceptors are needed. In this study, SrF_2_: Bi^3+^, Eu^3+^ phosphors are prepared using the solvothermal microwave-assisted method, and their blue/red dual emission is tuned by varying the Bi^3+^ and Eu^3+^ concentrations. SrF_2_: Bi^3+^, Eu^3+^ phosphors exhibit two emission bands in the wavelength ranges of 400–500 nm and 575–720 nm, which can be attributed to host emission, and ^5^D_0_  →  ^7^F*_J_* (*J* = 1, 2, 3, and 4) transitions of Eu^3+^, respectively. Our findings indicate that SrF_2_:Bi^3+^ and Eu^3+^ phosphors could be an excellent choice for increasing plant photosynthesis and growth in greenhouses.

## 2. Experimental

### 2.1. Synthesis of SrF_2_: x mol% Eu^3+^ (x = 1, 5, 10, 15, 20) and SrF_2_: 10 mol% Eu^3+^, y mol% Bi^3+^ (y = 5, 10, 15, 20, 30, 40, 50) Nanoparticles

Two sets of samples were synthesized as follows: (a) SrF_2_ doped with different europium ion concentrations such as 1, 5, 10, 15, and 20 mol% (Sr_1−*x*_Eu*_x_*F_2_ (*x* = 0.01, 0.05, 0.1, 0.15, 0.2), Table 1), and (b) SrF_2_ doped with 10 mol% of europium ions and 5, 10, 15, 20, 30, 40, and 50 mol% of bismuth ions (Sr_0.9−*y*_Eu_0.1_Bi*_y_*F_2_ (*y* = 0.05, 0.1, 0.15, 0.2, 0.3, 0.4, 0.5), Table 2). Additionally, one more sample of SrF_2_ doped with 20 mol% of bismuth ions was synthesized (SrF_2_:20Bi; exact amounts are given in Table S1 in Supplementary Materials).

The following chemicals for the synthesis of the desired samples were acquired and used as received: strontium (II) nitrate (Sr(NO_3_)_2_, Alfa Aesar, Ward Hill, MA, USA, 99%), europium (III) nitrate hexahydrate (Eu(NO_3_)_3_·6H_2_O, Alfa Aesar, 99.9%), bismuth (III) nitrate pentahydrate (Bi(NO_3_)_3_·5H_2_O, Sigma Aldrich, St. Louis, MO, USA, 98+%), sodium fluoride (NaF, Alfa Aesar, 99.9%), ethylene glycol (EG), and absolute ethanol (abs. EtOH).

In essence, strontium and europium nitrate in corresponding ratios (for the samples co-doped with bismuth ions, bismuth nitrate was added) were dissolved in ethylene glycol and stirred for 30 min. The next step is the addition of an EG solution of sodium fluoride, followed by another 30 min of stirring. The precursor mixture was then placed in the microwave reaction vessel, and the reaction conditions were as follows: (a) heating at 150 °C for 10 min, and (b) subsequently cooling to room temperature. The microwave experiments were carried out in an Anton-Paar microwave reactor (Monowave 400, Anton-Paar GmbH, Graz, Austria) in temperature control mode, utilizing a 30 mL Pyrex jar tightly sealed with a Teflon lid and stirred magnetically at 600 rpm. Each sample obtained through microwave synthesis was transferred into a centrifuge tube, centrifuged, and rinsed several times with abs. EtOH.

### 2.2. Characterization

Crystal structures of the obtained phosphors were investigated with an X-ray diffractometer (XRD) from Rigaku SmartLab, Tokyo, Japan (Cu-K_α1,2_ radiation, λ = 0.1540 nm) at room temperature. The experimental conditions for measurements were as follows: 2θ range of 20°–90°, with a step size of 0.02° and a counting time of 10°/min. Conclusions on the structural study (unit cell parameters, crystal coherence size, micro strain values, and data fit parameters) were attained using the built-in PDXL2 software v 2.1. The average particle size was calculated using ImageJ software V 1.8.0 (https://imagej.net/software/imagej/, accessed on 7 October 2024). The morphology was examined by a TESCAN MIRA3 field emission scanning electron microscope (FE-SEM), Brno, Czech Republic, with the samples coated using a thin layer of Au, and by transmission electron microscope (TEM) JEOL JEM1011, Tokyo, Japan operated at an accelerating voltage of 100 kV. The sample’s UV–VIS diffuse reflection spectrum was recorded using a Shimadzu UV-3600 UV-VIS-NIR spectrophotometer, Kyoto, Japan with BaSO_4_ used as the reflectance standard. Photoluminescent properties (PL) were studied utilizing a spectrofluorometric system FHR 1000 (Horiba Jobin-Yvon, Kyoto, Japan) equipped with a 300 grooves/mm grating and an ICCD detector (Horiba Jobin-Yvon 3771). Moreover, 265 and 405 nm LEDs from Ocean Optics (Tokyo, Japan) were used as excitation sources for the steady-state emission measurements. A 275 nm LED chip (CREE) with 100 mW optical power was used to excite a mixture of luminescent powder with transparent high-temperature inorganic binder (Cerambind 643-2 from Aremco, Van Nuys, CA, USA).

## 3. Results and Discussion

### 3.1. Structure and Morphology

**Figure 1:**
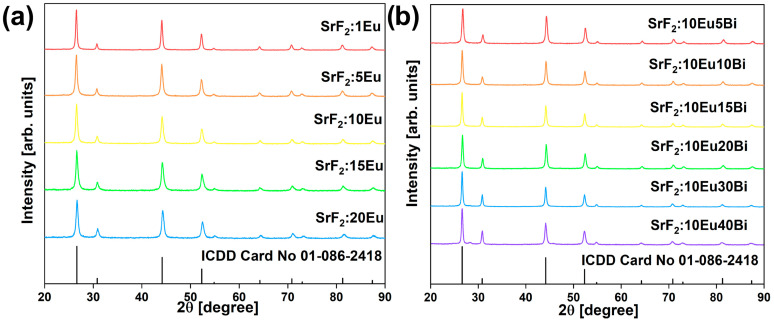
XRD patterns of (**a**) SrF_2_:*x*Eu (*x* = 1, 5, 10, 15, 20 mol%) and (**b**) SrF_2_:10Eu*y*Bi (*y* = 5, 10, 15, 20, 30, 40 mol%) samples presented with the ICDD card No. 01-086-2418.

Figure 1a,b show the X-ray pattern of SrF_2_:*x*Eu (*x* = 1, 5, 10, 15, 20 mol%) and SrF_2_: 10Eu*y*Bi (*y* = 5, 10, 15, 20, 30, 40 mol%) presented with the International Centre for Diffraction Data (ICDD) Card No. 01-086-2418. The X-ray diffraction examination of the synthesized samples proved a single-phase cubic structure with an -*3m* (225) space group (including the SrF_2_:20Bi sample; Figure S1a in the Supplementary Materials File). Traces of contaminations or other phase peaks were not observed in either set of samples, indicating that dopant Eu^3+^/Bi^3+^ ions were embedded into the SrF_2_ lattice. On the contrary, in the case of the SrF_2_:10Eu50Bi sample, there are additional peaks originating from a different phase, suggesting that the upper limit of dopant ion concentration in the made material is reached (Supplementary Materials Figure S1b).

Additionally, integrated PDXL2 software was used to obtain the values of the mean crystallite size and structural parameters presented in Table S2 (parameters for SrF_2_:*x*Eu (*x* = 1, 5, 10, 15, 20 mol%), SrF_2_:10Eu*y*Bi (*y* = 5, 10, 15, 20, 30, 40 mol%), and SrF_2_:20Bi sample). The initial parameters for the examination in PDXL2 software were collected from the reference [28]. The average crystallite size (CS) was calculated to be in the nanometer domain (~14–25 nm) for all the samples.

**Figure 2:**
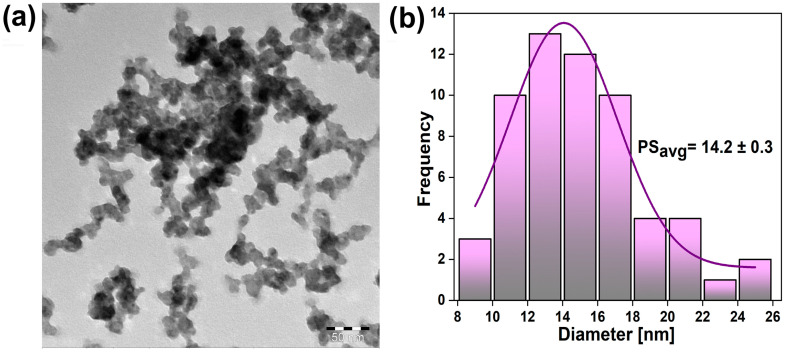
(**a**) TEM images of the representative SrF_2_:10Eu20Bi sample and (**b**) particle size distribution histogram.

A transmission electron microscopy (TEM) image of the representative SrF_2_:10Eu20Bi sample is shown in Figure 2a. Nanoparticles exhibit a pseudospherical shape, with the average particle size estimated to be 14.2 ± 0.3 nm (see the histogram fitted with a Gaussian distribution based on around 60 particles using the largest axis of the grain in Figure 2b). The calculated average particle size roughly equals the crystallite size obtained using X-ray diffraction.

### 3.2. Photoluminescence Properties

**Figure 3:**
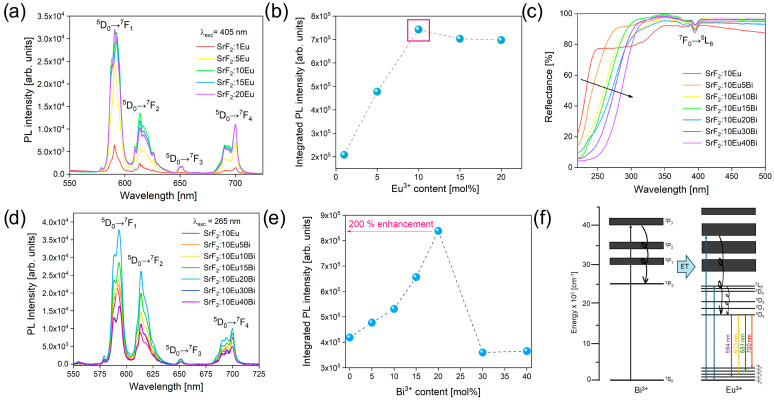
(**a**) Room temperature PL emission spectra under λ_exc_ = 405 nm of samples doped with only Eu^3+^ ions, (**b**) integrated intensity of PL spectra presented in (**a**), (**c**) diffuse reflectance spectra of samples doped with 10 mol% of Eu^3+^ ions and co-doped with Bi^3+^ ions, (**d**) room temperature PL emission spectra under λ_exc_ = 265 nm of co-doped samples, (**e**) integrated intensity of PL spectra presented in (**d**), and (**f**) schematic representation of the possible ET between Bi^3+^ and Eu^3+^ ions.

The photoluminescence (PL) emission spectra of the Eu^3+^-doped set of samples, namely, SrF_2_:1Eu, SrF_2_:5Eu, SrF_2_:10Eu, SrF_2_:15Eu, and SrF_2_:20Eu, recorded at room temperature are displayed in Figure 3a (λ_exc_ = 405 nm). All emissions correspond to *4f*–*4f* transitions of Eu^3+^ located at ~593 nm (^5^D_0_  → ^7^F_1_), ~614 nm (^5^D_0_  →  ^7^F_2_), ~651 nm (^5^D_0_  →  ^7^F_3_), and ~700 nm (^5^D_0_  →  ^7^F_4_). From the obtained emission spectra and integrated PL intensity (Figure 3b), it is evident that the sample SrF_2_:10Eu shows the highest emission intensity, and for this reason, it is chosen.

Figure 3c shows the diffuse reflectance spectra of Bi^3+^ co-doped SrF_2_:10Eu (Bi^3+^ mol% = 5, 10, 15, 20, 30, and 40) samples in the 220–500 nm wavelength range. The spectra show the absorption band at 394 nm corresponding to the Eu^3+^ transition from the ground state ^7^F_0_ to the upper level ^5^L_6_. In addition, it is observed that the UV band edge redshifts with an increase in Bi^3+^ content, indicating a strong absorption of Bi^3+^, which lies in the UV region [29,30,31].

Figure 3d shows PL emission spectra of Bi^3+^-co-doped samples in the 550–725 nm spectral region recorded at room temperature under 265 nm excitation. Europium ion emission intensity monotonically increases in the co-doped samples up to 20 mol% of Bi^3+^, while the further addition of Bi^3+^ decreases the emission intensity. The integrated emission intensity in the 550–725 nm wavelength range shows that the sample with the highest emission intensity—SrF_2_:10Eu20Bi—has twice as bright PL compared to the Bi-free SrF_2_:10Eu phosphor (Figure 3e). Energy transfer (ET) between bismuth (Bi^3+^) and europium (Eu^3+^) ions in inorganic hosts involves Bi^3+^ ions absorbing energy and transitioning from their ground state to excited states, followed by energy transfer to Eu^3+^ ions, exciting them from their ground state to higher state (Figure 3f). The efficiency of this process depends on factors such as the distance between ions, the spectral overlap between Bi^3+^ emission and Eu^3+^ absorption, and the properties of the host material. When excited, Eu^3+^ electrons radiatively return to the ground state and emit light at characteristic wavelengths, making this mechanism valuable for applications like LED displays, sensors, and solid-state lasers, and grasping these interactions enables tailoring of optical properties in materials development [32,33,34,35].

**Figure 4:**
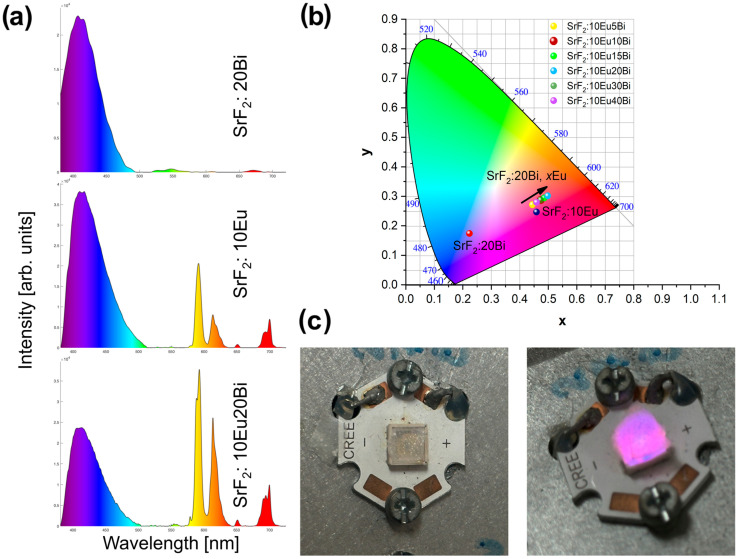
(**a**) The room temperature PL emission spectra of SrF_2_:20Bi, SrF_2_:10Eu, and SrF_2_:10Eu20Bi samples showing both blue- and red-light components in different ratios (λ_exc_ = 265 nm), (**b**) CIE chromaticity coordinates, and (**c**) a fabricated LED device displaying pinkish violet light.

Figure 4a shows the room temperature PL emission spectra (λ_exc_ = 265 nm) of SrF_2_:20Bi, SrF_2_:10Eu, and SrF_2_:10Eu20Bi samples in the 380–725 nm spectral region, showing both blue- and red-light components in different ratios. Emission spectra of SrF_2_:10Eu*y*Bi (*y* = 5, 10, 15, 30, and 40 mol%) samples in the 380–725 nm spectral region are presented in Supplementary Materials (Figure S2). Since the intense blue emission is present in both single-doped samples, it can be concluded that it originates from the host material. On account of the energy transfer from Bi^3+^ to Eu^3+^ [24,25], it is feasible to attain modifiable emission from blue to red in the SrF_2_:10Eu, Bi co-doped samples system by modulating Bi^3+^ content (5, 10, 15, 20, 30, and 40 mol%). Figure 4b shows the CIE chromaticity diagram for SrF_2_:20Bi, SrF_2_:10Eu, and SrF_2_:10Eu*y*Bi samples (*y* = 5, 10, 15, 20, 30, and 40 mol%). The CIE chromaticity coordinates move from blue for the SrF_2_:20Bi sample to pinkish for SrF_2_:10Eu, and orange-red areas with the increase in Bi^3+^ content in SrF_2_:10Eu*y*Bi, showing the color tunability in the produced series (CIE values are listed in Table 3).

Balancing blue- and red-light components is vital for optimizing plant health and maximizing yield in controlled environments. The integrated PL area in the 380–500 nm (blue) and 575–725 nm (red) wavelength range was used to determine the blue- and red-light emission portion. Table 3 shows that single-doped Bi^3+^ and Eu^3+^ SrF_2_ exhibit strong blue emissions corresponding to the host material; however, increasing Bi^3+^ concentration enhances Eu^3+^ red emission in Eu^3+^/Bi^3+^-activated samples. The highest red/blue emission portion (40.8:59.2) was found for the sample SrF_2_:10Eu20Bi.

Lastly, to demonstrate the potential application of the obtained material in LED fabrication, the powder sample with the highest emission intensity, SrF_2_:10Eu20Bi, was mixed with a ceramic binder and placed on top of a 275 nm near-UV chip. Photographs of the fabricated LED device, presented in Figure 4c, display strong pinkish violet light when the power supply is on.

## 4. Conclusions

The ability to convert UV into blue and red light in inorganic phosphors for LEDs in agricultural applications is essential to boost the photosynthesis of plants in greenhouses. We report tunable blue- and red-emitting Bi^3+^-co-doped SrF_2_:Eu^3+^ nanoparticles prepared via solvothermal microwave-assisted method. Upon 265 nm excitation, photoluminescence spectra show blue emission at around 400 nm originating from the host and characteristic orange ^5^D_0_ → ^7^F_1_, red ^5^D_0_ → ^7^F_2_ and ^5^D_0_ → ^7^F_3_, and deep red ^5^D_0_ → ^7^F_4_ Eu^3+^ emissions from Eu^3+^ ions.

In practice, most grow lights combine both red and blue wavelengths to offer a balanced spectrum that supports various stages of plant growth. For the best results, an adjustment of the ratio of red to blue light based on the plant’s specific needs and growth stage is needed. For example, plants like tomatoes, peppers, and orchids benefit from red light during their flowering and fruiting stages. For plants like strawberries or cucumbers, red light will support better fruit production. Leafy greens like lettuce, spinach, and kale thrive under blue light as it promotes healthy leaf growth. Also, blue light helps young seedlings develop strong, healthy leaves and stems, giving them a solid start. Tunable red and blue light reported here in Bi^3+^-co-doped SrF_2_:Eu^3+^ nanoparticles could ensure that plants receive the benefits of both types of light throughout their lifecycle.

## Figures and Tables

**Table 1:** Precursors for synthesis of 1 mmol of SrF_2_: *x* mol% Eu^3+^ (*x* = 1, 5, 10, 15, 20) samples.

Sample	Abbreviated Name	Sr(NO_3_)_2_ (g)	Eu(NO_3_)_3_·6H_2_O (g)	NaF (g)	EG (ml)
Sr_0.99_Eu_0.01_F_2_	SrF_2_:1Eu	0.2095	0.00446	0.0840	15
Sr_0.95_Eu_0.05_F_2_	SrF_2_:5Eu	0.2010	0.0223		
Sr_0.9_Eu_0.1_F_2_	SrF_2_:10Eu	0.1905	0.0446		
Sr_0.85_Eu_0.15_F_2_	SrF_2_:15Eu	0.1799	0.0669		
Sr_0.8_Eu_0.2_F_2_	SrF_2_:20Eu	0.1693	0.0892		

**Table 2:** Precursors for synthesis of 1 mmol of SrF_2_: 10 mol% Eu^3+^, *y* mol% Bi^3+^ (*y* = 5, 10, 15, 20, 30, 40, 50) samples.

Sample	Abbreviated Name	Sr(NO_3_)_2_ (g)	Eu(NO_3_)_3_·6H_2_O (g)	Bi(NO_3_)_3_·5H_2_O (g)	NaF (g)	EG (ml)
Sr_0.4_Eu_0.1_Bi_0.5_F_2_	SrF_2_:10Eu5Bi	0.1799	0.0446	0.0243	0.0840	15
Sr_0.8_Eu_0.1_Bi_0.1_F_2_	SrF_2_:10Eu10Bi	0.1693		0.0485		
Sr_0.75_Eu_0.1_Bi_0.15_F_2_	SrF_2_:10Eu15Bi	0.1587		0.0725		
Sr_0.7_Eu_0.1_Bi_0.2_F_2_	SrF_2_:10Eu20Bi	0.1481		0.0970		
Sr_0.6_Eu_0.1_Bi_0.3_F_2_	SrF_2_:10Eu30Bi	0.1269		0.1455		
Sr_0.5_Eu_0.1_Bi_0.4_F_2_	SrF_2_:10Eu40Bi	0.1058		0.1940		
Sr_0.4_Eu_0.1_Bi_0.5_F_2_	SrF_2_:10Eu50Bi	0.0846		0.2425		

**Table 3:** Blue and red emission portions for the SrF_2_:20Bi, SrF_2_:10Eu, and SrF_2_:10Eu*y*Bi (*x* = 5, 10, 15, 20, 30, and 40 mol%) samples.

Sample	% Blue	% Red	CIE (x, y) Coordinates
SrF_2_:20Bi	100.0	0.0	(0.223, 0.174)
SrF_2_:10Eu	85.0	15.0	(0.399, 0.247)
SrF_2_:10Eu5Bi	76.5	23.5	(0.444, 0.271)
SrF_2_:10Eu10Bi	71.6	28.4	(0.474, 0.288)
SrF_2_:10Eu15Bi	61.9	38.1	(0.486, 0.294)
SrF_2_:10Eu20Bi	59.2	40.8	(0.498, 0.301)
SrF_2_:10Eu30Bi	66.7	33.3	(0.473, 0.287)
SrF_2_:10Eu40Bi	73.1	26.9	(0.459, 0.279)

## Data Availability

Data are contained within the article and Supplementary Materials.

## Supplementary Materials

The following supporting information can be downloaded at: https://www.mdpi.com/article/10.3390/nano14201617/s1, Table S1: Exact amounts of precursors used for the synthesis of 1 mmol of SrF_2_:20Bi sample. Table S2: Selected structural parameters of the SrF_2_:*x*Eu (*x* = 1, 5, 10, 15, 20 mol%), SrF_2_:10Eu*y*Bi (*y* = 5, 10, 15, 20, 30, 40 mol%) and SrF_2_:20Bi nanopowders. Figure S1: XRD patterns of (a) SrF_2_:10Eu50Bi, and (b) SrF_2_:20Bi samples. The diffraction peaks are indexed according to the ICDD card No. 01-086-2418. Figure S2: The room temperature PL emission spectra of SrF_2_:10Eu*x*Bi (*x* = 5, 10, 15, 30 and 40 mol%) samples measured in 380–725 nm spectral range showing both blue and red-light components in different ratios (λ_exc_ = 265 nm).
