# Supplementary material for: Tuneable Red and Blue Emission of Bi3+-Co-Doped SrF2:Eu3+ Nanophosphors for LEDs in Agricultural Applications
Source: Nanomaterials (Basel). 2024 Oct 10;14(20):1617. doi: 10.3390/nano14201617 (PMC11510959; doi:10.3390/nano14201617)
Supplement: Supplementary file 1 [file nanomaterials-14-01617-s001.zip › nanomaterials-3238555-supplementary.pdf]

Supporting information file

Table S1 Exact amounts of precursors used for the synthesis of 1mmol of SrF_2:20Bi sample

	Abbreviated name	$Sr(NO_3)_2$ (g)	$Bi(NO_3)_3$ x5H_2O(g)	NaF (g)	EG (ml)
**$Sr_{0.8}Bi_{0.2}F_2$**	SrF_2:20Bi	0.1693	0.0970	0.0840	15

Table S2 Selected structural parameters of the SrF_2:*x*Eu (*x*= 1, 5, 10, 15, 20 mol%), SrF_2:10Eu*y*Bi (*y*= 5, 10, 15, 20, 30, 40 mol%) and SrF_2:20Bi nanopowders.

ICDD card 01-086-2418	a=b=c (Å)	CS (Å)	Strain	GOF	Rwp (%)	Rp (%)	Re (%)
**SrF_2:1Eu**	5.7970(2)	183.5(11)	0.14(6)	1.0426	7.65	5.77	7.34
**SrF_2:5Eu**	5.7979(3)	131.2 (11)	0.24(9)	1.0247	7.38	5.77	7.20
**SrF_2:10Eu**	5.79128(14)	150.2(5)	0.15(3)	1.0261	7.42	5.74	7.23
**SrF_2:15Eu**	5.7973(5)	117.9 (10)	0.19(9)	1.0567	7.38	5.78	6.98
**SrF_2:20Eu**	5.7838(5)	123.7 (13)	0.14(3)	1.0936	7.50	5.98	6.86
**SrF_2:10Eu5Bi**	5.7907(4)	149.0 (18)	0.23(11)	1.1974	8.73	6.79	7.29
**SrF_2:10Eu10Bi**	5.7914(3)	188(2)	0.06(10)	1.1738	8.64	6.59	7.36
**SrF_2:10Eu15Bi**	5.7942(4)	209(2)	0.08(7)	1.4142	10.68	7.96	7.55
**SrF_2:10Eu20Bi**	5.7918(5)	178(3)	0.123(5)	1.4067	10.86	8.10	7.72
**SrF_2:10Eu30Bi**	5.8023(5)	250(18)	0.220(9)	1.7336	12.84	9.49	7.40
**SrF_2:10Eu40Bi**	5.8080(13)	201(5)	0.23(8)	1.6523	12.63	9.14	7.87
**SrF_2: 20Bi**	5.8011(3)	250.5(16)	0.241(5)	1.1840	8.75	6.63	7.39

* Rwp—the weighted profile factor; ** Rp—the profile factor; *** Re—the expected weighted profile factor; GOF—the goodness of fit.

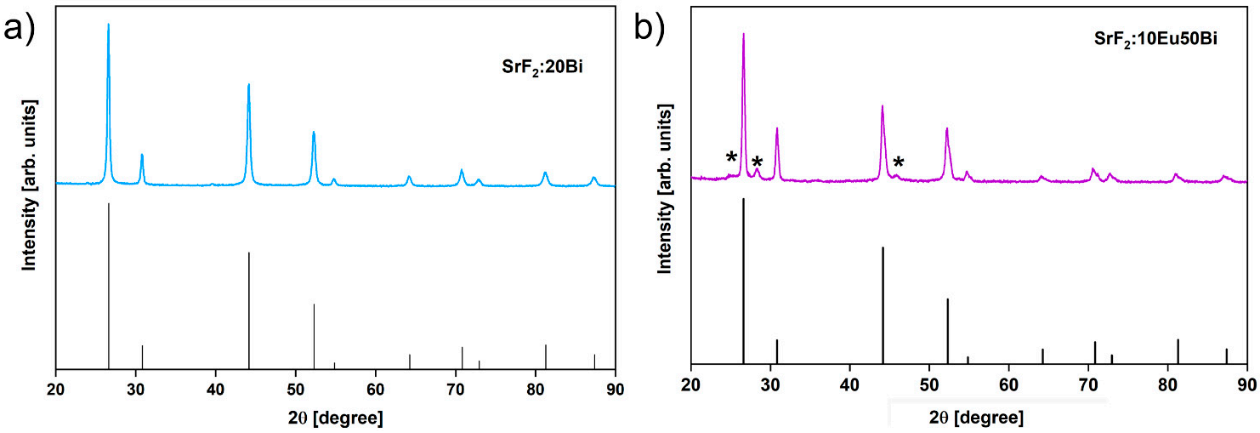

Figure S1 Figure 1 XRD patterns of a) SrF_2:10Eu50Bi, and b) SrF_2:20Bi samples. The diffraction peaks are indexed according to the ICDD card No. 01-086-2418.

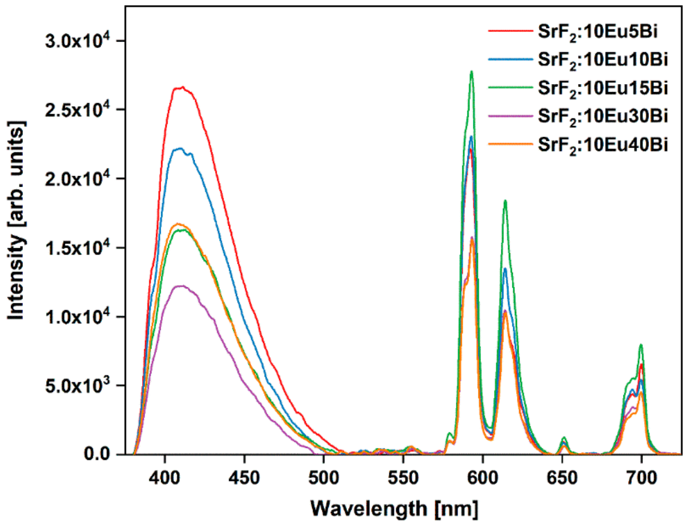

Figure S2 The room temperature PL emission spectra of SrF_2:10Eu*x*Bi (*x* = 5, 10, 15, 30 and 40 mol%) samples measured in 380–725 nm spectral range showing both blue and red-light components in different ratios (λ_{exc} = 265 nm).
